# The immune microenvironment and population heterogeneity in myasthenia gravis: implications for precision therapeutics

**DOI:** 10.3389/fimmu.2026.1932949

**Published:** 2026-08-20

**Authors:** Xia Xue, Chang Liu, Chunjing Qiu, Simeng Liu, Fei Liu, Ya Li

**Affiliations:** State Key Laboratory of Metabolic Dysregulation and Prevention and Treatment of Esophageal Cancer, Henan Key Laboratory for Helicobacter pylori and Digestive Tract Microecology, The Fifth Affiliated Hospital of Zhengzhou University, Zhengzhou, China

**Keywords:** immune microenvironment, myasthenia gravis, novel markers, population genetics, precision treatments

## Abstract

Myasthenia gravis (MG) is a chronic autoimmune disorder of the neuromuscular junction characterized by fluctuating skeletal muscle weakness. Although the pathogenic roles of autoantibodies targeting the acetylcholine receptor (AChR), muscle-specific kinase (MuSK), and certain neuromuscular junction proteins have been well established, increasing evidence indicates that MG onset, progression, and therapeutic response can be shaped by the complex interactions within diverse immune microenvironments. Particularly, population heterogeneity, including differences in antibody subtype, age, sex, genetic susceptibility, ethnicity, thymic pathology, and potential confounders related to differences in healthcare conditions, strongly contributes to variability in its clinical manifestations and targeted therapies. This review discusses current evidence on the roles of the immune microenvironment and individual heterogeneity in shaping the pathogenesis and treatment landscape of MG. Along with the contributions of immunophenotyping, multi-omics techniques, single-cell and spatial transcriptomics, and biomarker discovery in improving our understanding of MG mechanisms, we also evaluated the implications of immune diversity in various populations for established therapies, as well as emerging immune-modulating approaches. This review highlights current knowledge gaps and future research priorities, emphasizing the transition from phenotype-based disease classification toward immune endotype-driven precision medicine within population heterogeneity.

## Introduction

Myasthenia gravis (MG) is a chronic autoimmune neuromuscular disease that disrupts communication between nerves and muscles (1, 2). It is characterized by fluctuating skeletal muscle weakness and fatigability resulting from impaired neuromuscular transmission (3, 4). MG is primarily mediated by pathogenic autoantibodies directed against proteins involved in synaptic signal transmission, most commonly the acetylcholine receptor (AChR), muscle-specific kinase (MuSK), and low-density lipoprotein receptor-related protein 4 (LRP4) (5). These autoantibodies disrupt the structure and function of the postsynaptic membrane through distinct immunopathological mechanisms, including complement-mediated tissue injury, receptor internalization, and interference with receptor clustering (6–9). Despite sharing a common clinical phenotype, MG shows high heterogeneity in disease severity, progression, and treatment response, highlighting its complexity beyond a conventional autoimmune disorder (6, 10–12).

**Figure 1:**
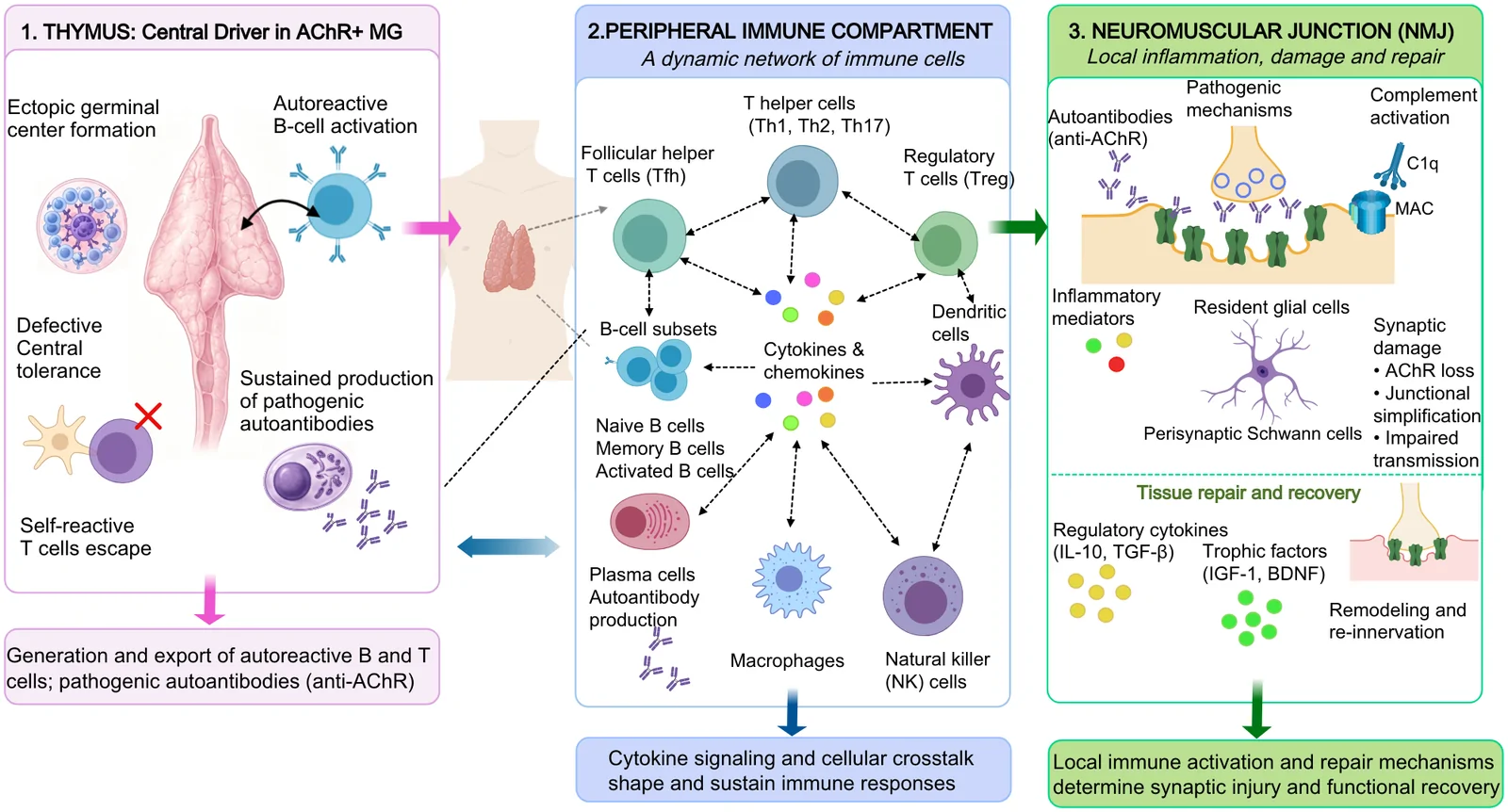
Multicompartmental immunopathogenesis of acetylcholine receptor-positive myasthenia gravis (AChR^+^MG). The pathogenesis of AChR^+^ MG involves coordinated immune dysregulation across three interconnected compartments: the thymus, peripheral immune system, and neuromuscular junction (NMJ). (1) Thymic abnormalities, including ectopic germinal center formation and defective central tolerance, permit the escape of autoreactive T cells and promote autoreactive B-cell activation. These processes sustain the generation of plasma cells and the production of pathogenic anti-acetylcholine receptor (AChR) autoantibodies, which are exported alongside autoreactive lymphocytes into the peripheral circulation. (2) A dynamic network of immune cells, including T helper (Th1, Th2, Th17), follicular helper T (Tfh), regulatory T (Treg), B-cell subsets, plasma cells, dendritic cells, macrophages, and natural killer (NK) cells, communicates through cytokines and chemokines to amplify or regulate autoreactive immune responses. Cellular crosstalk within this compartment supports continued autoantibody production and shapes disease activity. (3) Circulating anti-AChR autoantibodies bind postsynaptic AChRs, triggering pathogenic mechanisms including complement activation, membrane attack complex (MAC) formation, and local inflammatory responses. These processes lead to AChR loss, simplification of the postsynaptic membrane, impaired neuromuscular transmission, and synaptic dysfunction. Resident glial cells, including perisynaptic Schwann cells, contribute to both inflammatory signaling and tissue repair. Recovery is promoted through regulatory cytokines (e.g., IL-10 and TGF-β), trophic factors (e.g., IGF-1 and BDNF), and mechanisms of synaptic remodeling and reinnervation. Bidirectional communication between the thymus, peripheral immune system, and NMJ drives disease progression while also influencing repair and functional recovery (26–30).

The pathogenesis of MG has been explained by dysregulated humoral immunity. However, accumulating evidence indicates that disease development is driven by intricate interactions within the immune microenvironment (13–16). The thymus plays a central role in patients with AChR-positive MG, serving as a site of ectopic germinal center formation, autoreactive B-cell activation, defective central tolerance, and sustained production of pathogenic autoantibodies (17). Besides the thymus, the peripheral immune compartment comprises a dynamic network of B-cell subsets, T helper cells, regulatory T cells, follicular helper T cells, dendritic cells, macrophages, natural killer cells, and plasma cells that collectively shape immune responses through cytokine signaling and cellular crosstalk (18–24). At the neuromuscular junction, local complement activation, inflammatory mediators, resident glial cells, and tissue repair mechanisms further influence synaptic damage and recovery (25). These interconnected immune niches constitute a complex immunological ecosystem that determines disease initiation, persistence, and clinical progression (**Figure 1**).

The immune microenvironmental diversity has coincided with the emergence of substantial population heterogeneity in MG. Clinical and biological differences are evident across antibody subtypes, age at onset, sex, ethnicity, genetic background, thymic pathology, and environmental exposures (31–37). Early-onset AChR-positive MG is frequently associated with thymic hyperplasia and a predominance of females, while late-onset disease often develops in the context of immunosenescence and chronic low-grade inflammation (38, 39). In contrast, MuSK-positive MG shows distinct immunological features characterized by IgG4 autoantibodies, limited complement activation, and an enhanced response to B-cell-depleting therapies (40, 41). The genetic diversity further contributes to differences in MG prevalence, clinical manifestations, autoantibody profiles, and therapeutic outcomes, underscoring the limitations of a “one-size-fits-all” treatment approach (38). Although these variations may reflect genuine differences in genetic susceptibility, environmental exposures, and immune regulation, they are also influenced by disparities in healthcare access, diagnostic criteria, referral patterns, disease awareness, and study methodology. Therefore, disentangling biological heterogeneity from methodological and healthcare-related confounders remains essential for accurately defining population-specific disease characteristics and for advancing precision medicine in MG.

The expanding therapeutic landscape of MG emphasizes the significance of understanding disease under population heterogeneity. Conventional immunosuppressive therapies, including corticosteroids and steroid-sparing agents, remain the cornerstone of treatment but are associated with considerable adverse effects and variable efficacy. More recently, targeted biological therapies, such as complement inhibitors, neonatal Fc receptor (FcRn) antagonists, and B-cell-directed therapies, have transformed the management of selected patient populations (14, 42). For instance, FcRn drives the recycling of IgG antibodies, and its inhibitors deplete pathogenic IgG autoantibodies without broad immunosuppression, providing an efficient treatment for MG. Modulating B-cells offers a more sustained approach to reducing autoantibody production, which works particularly transformatively for patients with MuSK antibody-positive MG, among whom most complement inhibitors are ineffective. The resulting disorganization of the postsynaptic apparatus produces severe neuromuscular transmission defects despite the relative absence of complement-mediated tissue injury (**Table 1**). These mechanistic differences somehow explain that patients with MuSK-positive MG often present with prominent bulbar and respiratory muscle involvement, respond less favorably to acetylcholinesterase inhibitors, and frequently demonstrate excellent clinical improvement following B-cell depletion therapy.

**Table 1:** Immune tolerance defects and subtype-specific pathogenic mechanisms in myasthenia gravis.

Pathogenic process	AChR-positive MG	MuSK-positive MG	Biological significance
Breakdown of immune tolerance	Failure of central tolerance within the thymus permits survival of autoreactive T cells, impaired peripheral tolerance allows activation of autoreactive B cells.	Thymic abnormalities are generally absent or minimal.	Represents the initiating event that enables production of pathogenic autoantibodies.
Thymic pathology	Thymic follicular hyperplasia with ectopic germinal centers, thymoma in approximately 10–15% of cases.	Thymus is histologically normal and rarely contains ectopic germinal centers.	Highlights fundamentally different sites of immune activation between MG subtypes
Dominant immune cells	Tfh cells, germinal center B cells, plasma cells, follicular dendritic cells, autoreactive CD4^+^ T cells, and dysfunctional Tregs cooperate to sustain autoantibody production.	Short-lived plasmablasts and memory B cells producing IgG4 antibodies, T-cell help remains important but germinal-center responses are less prominent.	Different cellular networks influence therapeutic responses to B-cell-targeted treatments.
Immune regulatory defects	Reduced Treg suppressive activity, increased inflammatory cytokines, defective negative selection of autoreactive T cells.	Dysregulated B-cell differentiation and persistent autoreactive antibody production despite limited thymic involvement.	Loss of immune regulation perpetuates chronic autoimmunity.
Pathogenic autoantibody	Mainly IgG1 and IgG3 antibodies against AChR.	Predominantly IgG4 antibodies against MuSK.	Antibody subclass determines the dominant mechanism of tissue injury.
Primary molecular mechanism	Complement activation, receptor cross-linking, antigenic modulation, and accelerated internalization of AChRs.	Disruption of MuSK–LRP4 signaling, preventing agrin-induced AChR clustering without substantial complement activation.	Produces structurally distinct forms of neuromuscular junction dysfunction.
Complement involvement	Strong activation of the classical complement pathway with membrane attack complex deposition.	Minimal complement activation because IgG4 poorly activates complement.	Complement inhibitors are effective primarily in AChR-positive MG.
Neuromuscular junction pathology	Simplification of postsynaptic folds, loss of AChRs, complement-mediated membrane injury.	Relatively preserved postsynaptic membrane architecture but marked dispersal of AChR clusters.	Different structural abnormalities result in impaired neuromuscular transmission.
Typical clinical phenotype	Generalized weakness with fluctuating ocular and limb involvement; variable disease severity.	More severe bulbar, facial, neck, and respiratory weakness; muscle atrophy may occur.	Reflects distinct pathogenic mechanisms affecting synaptic organization.

Nevertheless, notable variability in clinical response persists, even among patients with similar antibody levels, suggesting that additional immunological factors within the immune microenvironment influence therapeutic effectiveness (43–45). This review examines the current understanding of the immune microenvironment and population heterogeneity in MG and discusses their implications for disease pathogenesis, biomarker discovery, and therapeutic intervention. We synthesize evidence describing the immunological interactions within the thymus, peripheral immune system, and neuromuscular junction while highlighting how differences in antibody subtype, demographic characteristics, genetic susceptibility, and immune profiles influence clinical outcomes and responses to treatment. Furthermore, we discuss emerging technologies and future potential directions, as well as their limits, that may facilitate the transition from conventional phenotype-based management toward immune endotype-driven precision medicine, eventually improving personalized care for patients with MG.

## Complement activation and inflammatory signaling

Complement activation is one of the most extensively characterized mechanisms of tissue injury in AChR-positive MG. Binding of IgG1 and IgG3 autoantibodies initiates the classical complement pathway through C1q recognition, resulting in sequential activation of C4, C2, and C3 before culminating in cleavage of complement component C5 (46). This generates the potent inflammatory mediator C5a and the membrane attack complex (C5b-9), which damages the postsynaptic membrane (47). Complement-mediated injury destroys acetylcholine receptors and also recruits inflammatory cells and enhances local cytokine production, creating a self-perpetuating inflammatory microenvironment (25). Experimental studies have demonstrated that inhibition of terminal complement activation preserves neuromuscular junction architecture and improves muscle strength, findings that have translated successfully into targeted clinical therapies (48).

Autoantibodies are the defining immunopathological hallmark of MG. However, the mechanisms by which they disrupt neuromuscular transmission vary according to antigen specificity. The antibody binding activates the classical complement cascade, leading to the formation of the membrane attack complex (MAC), destruction of the postsynaptic membrane, and simplification of the junctional folds (49). Complement-mediated injury substantially reduces the safety factor for neuromuscular transmission and is a major determinant of muscle weakness. Also, bivalent antibodies cross-link adjacent AChRs, accelerating receptor internalization and lysosomal degradation. This process decreases receptor density on the postsynaptic membrane and compromises synaptic signaling. Moreover, autoantibodies directly block acetylcholine binding sites, thereby interfering with receptor activation and reducing the efficiency of neurotransmission (14). The combined effects of complement activation, receptor loss, and functional blockade produce the characteristic fatigable weakness associated with AChR-positive MG. Beyond complement, numerous cytokines and chemokines contribute to disease pathogenesis. Elevated concentrations of IL-6, IL-17, interferon-γ (IFN-γ), tumor necrosis factor-α (TNF-α), B-cell activating factor (BAFF), a proliferation-inducing ligand (APRIL), and CXCL13 have been detected in patients with active MG. These molecules promote B-cell survival, plasma-cell differentiation, T-cell activation, and leukocyte recruitment, thereby reinforcing chronic immune activation. The cytokine milieu also varies among MG subtypes, suggesting that inflammatory networks contribute to both disease heterogeneity and therapeutic responsiveness (50).

## Genetic and environmental influences

**Figure 2:**
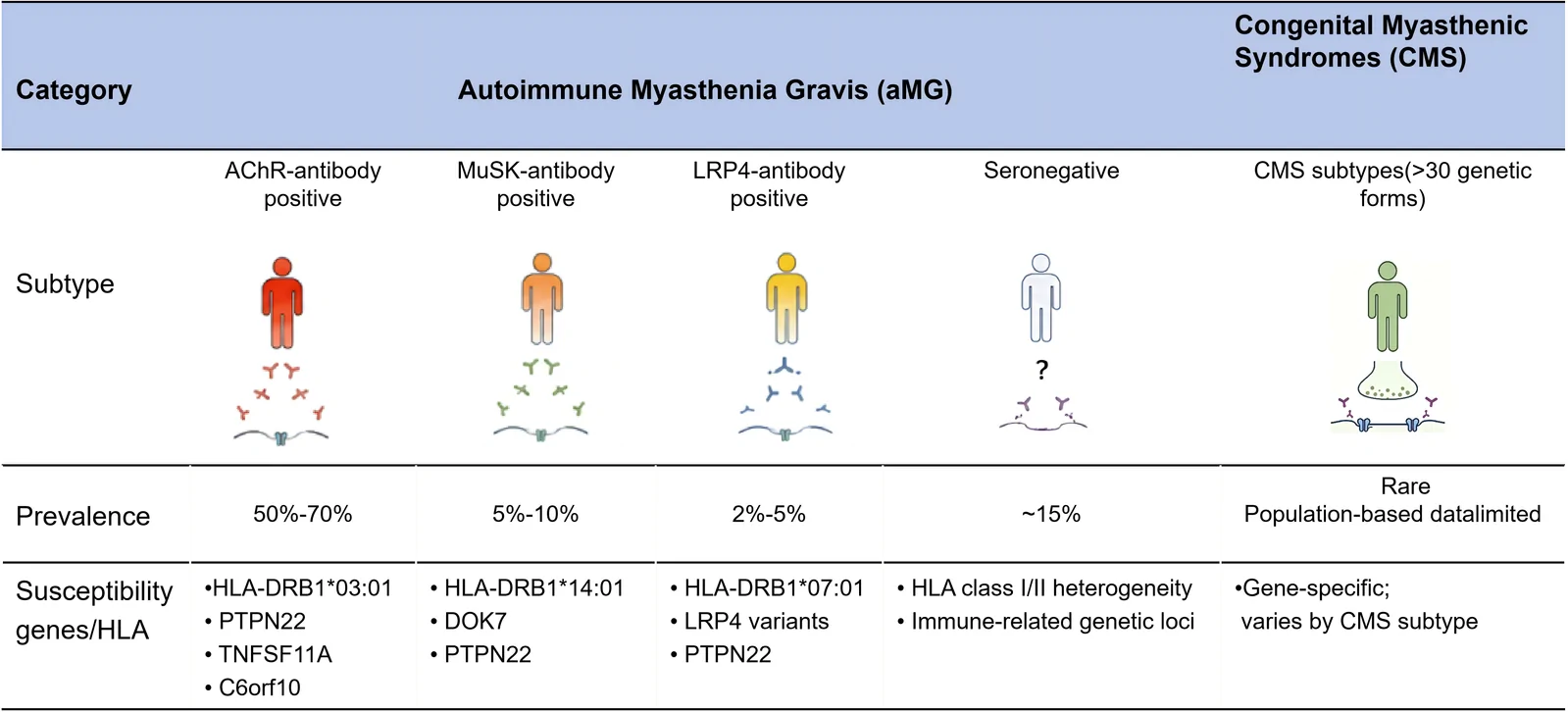
The distinct genetic associations and heterogeneous pathobiology across myasthenia gravis (MG) subtypes. Schematic comparison of autoimmune myasthenia gravis (aMG) and congenital myasthenic syndromes (CMS). Autoimmune MG is classified by antibody status (AChR, MuSK, LRP4, and seronegative), with the approximate prevalence and representative genetic susceptibility loci shown for each subtype. CMS comprises more than 30 genetically distinct inherited disorders with subtype-specific causal genes (53–57).

The development of MG reflects a complex interplay between inherited susceptibility and environmental triggers (51). Multiple human leukocyte antigen (HLA) alleles have been associated with MG risk, particularly among early-onset AChR-positive MG, while polymorphisms affecting immune regulatory genes may influence immune tolerance and disease susceptibility. Genetic susceptibility contributes substantially to MG development but is insufficient to cause disease independently. Genome-wide association studies have identified numerous susceptibility loci involved in immune regulation, including HLA genes, CTLA4, PTPN22, TNFRSF11A, TNIP1, and genes associated with cytokine signaling and lymphocyte activation (52). The strongest genetic associations involve the HLA region, which plays a central role in antigen presentation and T-cell selection. Different HLA haplotypes are associated with specific clinical phenotypes, including early-onset MG, MuSK-positive disease, and thymoma-associated MG. These observations suggest that genetic background influences disease susceptibility as well as immunological endotypes (**Figure 2**) (53). Genetic polymorphisms may additionally affect complement activation, Fc receptor function, cytokine production, and responses to immunotherapy. For example, variations influencing complement regulation could theoretically modify responsiveness to complement inhibitors, whereas polymorphisms affecting B-cell activation pathways may alter the effectiveness of B-cell-targeted therapies. Although many of these associations remain under investigation, integration of genomic information into clinical practice may eventually facilitate individualized treatment selection.

Environmental factors are increasingly recognized as important modifiers of MG risk and progression in genetically susceptible individuals (31). Rather than acting independently, these exposures interact with host genetic susceptibility and the immune microenvironment to influence the initiation and perpetuation of autoimmune responses. Viral infections, alterations in the gut microbiome, chronic inflammation, smoking, psychological stress, hormonal changes, and exposure to certain medications have all been implicated as potential triggers of autoimmune activation (58, 59). However, the relative contribution of individual environmental factors likely varies among MG subtypes and patient populations, reflecting differences in genetic background, age, sex, geographic location, and cumulative lifetime exposures. Among these factors, the intestinal microbiome has emerged as a key regulator of immune homeostasis. Growing evidence indicates that patients with MG exhibit alterations in gut microbial composition and reduced microbial diversity compared with healthy individuals. A notable reduction has been reported in MG patients in beneficial, short-chain fatty acid (SCFA)-producing bacterial genera, such as *Clostridium*, *Faecalibacterium*, and *Bifidobacterium*. While MG patients show elevated levels of opportunistic pathogens and pro-inflammatory bacteria, including *Streptococcus*, *Escherichia-Shigella*, and *Bacteroidetes*. Such dysbiosis may impair intestinal barrier integrity, alter microbial metabolite production, and disrupt the balance between regulatory T cells and pro-inflammatory Th17 cells, thereby promoting aberrant cytokine production and loss of peripheral immune tolerance (60). Microbiome-derived metabolites, particularly SCFA, are increasingly recognized for their role in maintaining regulatory immune networks, suggesting that perturbations in these pathways may contribute to chronic immune activation in MG. Nevertheless, whether microbiome alterations represent a primary pathogenic mechanism or a secondary consequence of disease, immunosuppressive therapy, dietary habits, or other environmental influences remains incompletely understood (61).

In parallel, lifestyle-related factors further contribute to interindividual variability in disease expression. Physical inactivity, obesity, poor sleep quality, and chronic psychological stress may amplify systemic inflammation, impair neuromuscular function, and exacerbate fatigue, thereby increasing disease burden even in patients with stable immunological activity. Conversely, regular exercise, individualized rehabilitation, adequate nutritional support, optimized sleep, and effective management of metabolic and psychological comorbidities may improve physical function, fatigue, and health-related quality of life, despite exerting only indirect effects on autoimmune mechanisms (62). Importantly, environmental exposures are not uniformly distributed across populations. Geographic variation in diet, infection burden, pollution, socioeconomic status, healthcare access, and cultural practices may contribute to differences in immune phenotypes and clinical outcomes, although these associations are frequently confounded by disparities in study design, case ascertainment, and diagnostic practices. Integrating environmental, microbial, genetic, and immunological data through multi-omics approaches will therefore be essential for identifying biologically relevant disease endotypes and developing precision therapeutic strategies tailored to individual patients with MG.

## The immune microenvironment in myasthenia gravis

As we mentioned, the microenvironment in MG extends across multiple anatomical compartments, including the thymus, peripheral lymphoid tissues, circulation, and neuromuscular junction, each contributing distinct but interconnected immunological processes that influence disease onset, progression, and therapeutic response. Notably, these immune niches do not function independently. Continuous communication among central and peripheral immune compartments creates a self-sustaining autoimmune network that promotes autoreactive lymphocyte survival, pathogenic antibody production, complement activation, and chronic inflammation (63, 64).

**Figure 3:**
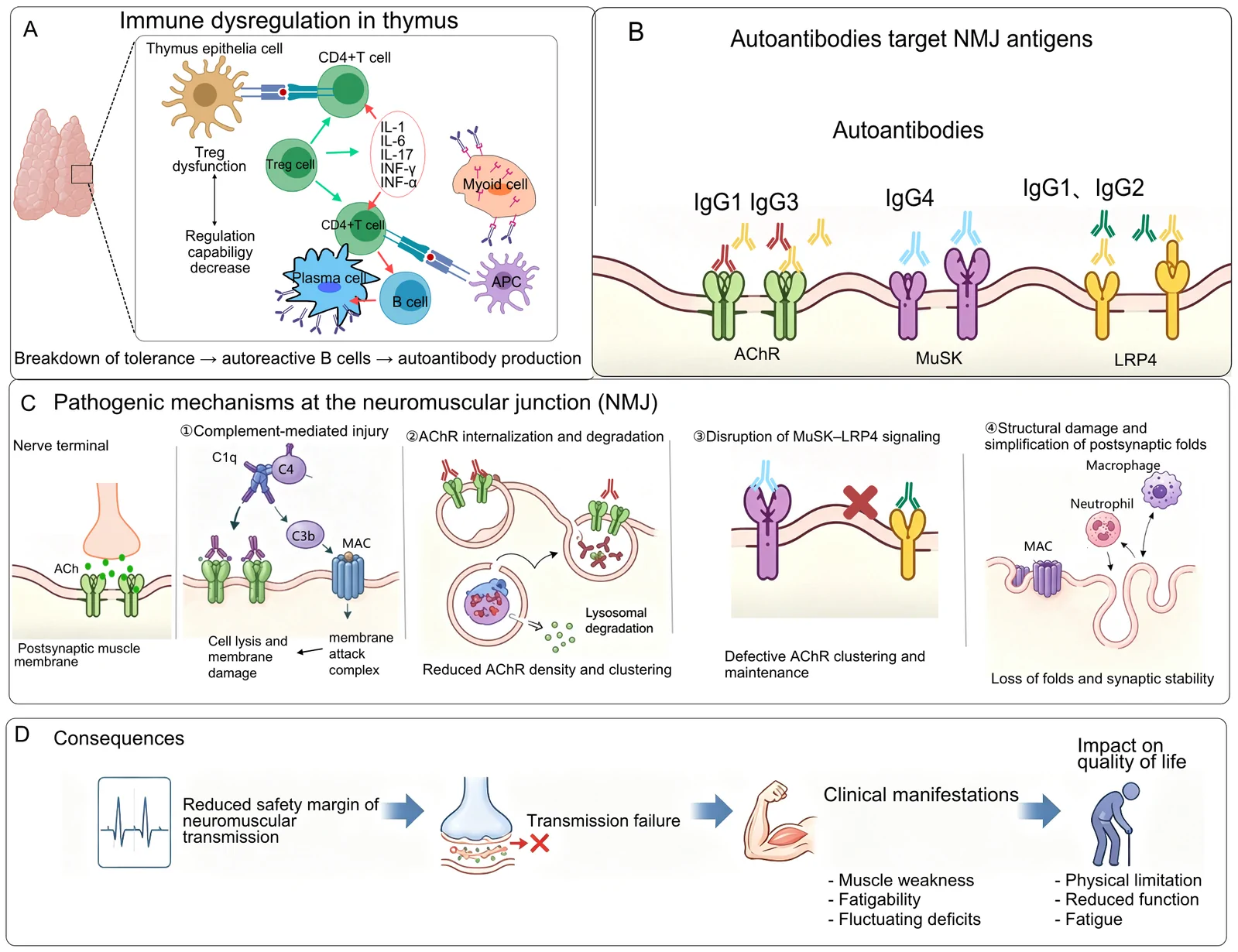
The pathophysiological mechanism of myasthenia gravis (MG). **(A)** Proposed mechanisms underlying immune dysregulation in MG. Thymic abnormalities promote activation of autoreactive CD4^+^T cells, impaired regulatory T-cell function, and B-cell differentiation into plasma cells that produce pathogenic autoantibodies, supported by pro-inflammatory cytokines and antigen-presenting cells (APCs). **(B)** The distinct roles of different IgG subclasses in MG. **(C)** Pathogenic effects of autoantibodies at the neuromuscular junction (NMJ). AChR, MuSK, and LRP4 autoantibodies disrupt neuromuscular transmission through distinct mechanisms, including loss of AChR density and function, impaired MuSK signaling, and disruption of the LRP4-MuSK interaction. Complement activation and membrane attack complex (MAC) formation, primarily in AChR-antibody-positive disease, result in postsynaptic membrane damage and simplification of junctional folds, leading to reduced endplate potentials, transmission failure, and the characteristic muscle weakness and fatigability of MG. **(D)** The phenotypic consequences of MG.

The thymus is the primary site of T-cell development and central immune tolerance, where autoreactive T lymphocytes are normally eliminated through positive and negative selection. In AChR antibody-positive MG, however, this highly regulated environment undergoes profound structural and functional remodeling, transforming into a chronic inflammatory niche that supports autoimmune responses (14). Thymic epithelial cells also play a pivotal role in maintaining immune tolerance by presenting self-antigens to developing T cells (65). In MG, abnormalities in thymic epithelial cell function, altered expression of the AIRE gene, and persistent inflammatory signaling impair negative selection, allowing autoreactive T cells to escape into the peripheral circulation (66). These escaped T cells subsequently support autoreactive B cells, perpetuating autoantibody production (67). Beyond producing pathogenic autoantibodies, they function as efficient antigen-presenting cells capable of activating autoreactive CD4^+^ T cells. T-cell subsets exert equally important regulatory functions. Follicular helper T cells facilitate germinal center formation through IL-21 secretion and expression of CD40 ligand, thereby promoting B-cell maturation and affinity maturation. In contrast, Treg cells exhibit reduced suppressive capacity, permitting persistent activation of autoreactive lymphocytes. Increased Th17 cells and elevated IL-17 concentrations further amplify inflammation by recruiting neutrophils, stimulating cytokine production, and enhancing B-cell activation (**Figure 3**) (68). These alterations create an immune environment skewed toward autoimmunity rather than immune tolerance.

Recent advances in single-cell RNA sequencing have demonstrated remarkable diversity among circulating immune cell populations in MG. Distinct transcriptional signatures have been identified in B cells, T cells, and monocytes, suggesting that disease activity reflects coordinated alterations across multiple immune lineages rather than isolated dysfunction of individual cell types (15). Such findings support the concept of MG as a systems-level immune disorder. Resident Schwann cells, traditionally regarded as supportive glial cells, are increasingly recognized as active participants in neuromuscular homeostasis. Following injury, Schwann cells secrete neurotrophic factors that facilitate synaptic maintenance and regeneration while interacting with infiltrating immune cells through cytokine signaling (69). Impaired communication between Schwann cells and immune cells may compromise neuromuscular repair, contributing to persistent weakness despite reduction in circulating autoantibody levels (70). Macrophages exhibit functional plasticity within the NMJ. Pro-inflammatory (M1-like) macrophages promote cytokine release and tissue injury, whereas anti-inflammatory (M2-like) macrophages facilitate debris clearance, extracellular matrix remodeling, and tissue repair (71). The balance between these macrophage phenotypes may influence disease severity and recovery following treatment, although this remains an area of active investigation. Extracellular matrix components within the synaptic basal lamina also influence immune responses by regulating complement deposition, cell adhesion, and tissue remodeling (72, 73). Alterations in matrix composition may affect both synaptic stability and immune cell recruitment, highlighting the structural environment as an important determinant of disease progression. NMJ is thus not merely a passive target of autoimmune attack but an active participant in the inflammatory process.

## Crosstalk between immune niches

One of the defining characteristics of the MG immune microenvironment is the continuous communication among anatomical compartments. Autoreactive T cells generated within the thymus migrate to peripheral lymphoid organs, where they activate B cells and promote autoantibody production. Circulating antibodies subsequently target the neuromuscular junction, triggering complement activation and local inflammation (74). Tissue injury at the NMJ releases additional self-antigens and inflammatory mediators, further stimulating peripheral immune responses and reinforcing the autoimmune cycle (75). This bidirectional communication highlights why interventions directed at a single immune component may not completely suppress disease activity (**Table 2**). For example, depletion of circulating B cells does not eliminate long-lived plasma cells, while complement inhibition prevents tissue injury without directly reducing autoantibody production (76). Likewise, FcRn inhibitors lower circulating IgG concentrations but do not address persistent immune dysregulation within thymic or peripheral tissues. These observations illustrate the importance of considering MG as an interconnected immune ecosystem rather than a disease driven by a single pathogenic pathway (77).

**Table 2:** Inter-organ immune communication and implications for precision medicine in myasthenia gravis.

Immune ecosystem component	Key biological events	Impact on disease progression
Thymic immune niche	Escape of autoreactive T cells following defective central tolerance, activation of germinal center responses in AChR-positive MG	Initiates and sustains autoreactive B-cell responses
Peripheral immune compartment	Continuous interactions among T cells, B cells, plasma cells, dendritic cells, macrophages, NK cells, and cytokine networks	Amplifies autoantibody production and maintains chronic inflammation
Neuromuscular junction (NMJ)	Autoantibody binding triggers complement activation, inflammatory signaling, and synaptic injury	Causes impaired neuromuscular transmission and muscle weakness
Feedback between immune compartments	Tissue damage releases self-antigens and inflammatory mediators that reactivate peripheral immune responses	Establishes a self-perpetuating autoimmune loop
Long-lived plasma cells	Persistent production of pathogenic antibodies despite depletion of circulating B cells	Maintains disease activity after conventional B-cell depletion
FcRn-mediated IgG recycling	FcRn protects IgG from lysosomal degradation, prolonging the half-life of pathogenic autoantibodies	Sustains circulating autoantibody levels
Immune microenvironment heterogeneity	Differences in immune-cell composition, cytokine profiles, complement activity, and tissue inflammation among patients	Contributes to variability in disease severity, progression, and treatment
Systems immunology technologies	Single-cell transcriptomics, mass cytometry, proteomics, metabolomics, spatial transcriptomics, and multidimensional immune profiling	Enables comprehensive characterization of immune ecosystems and identification of pathogenic pathways
Precision medicine framework	Integration of immune profiling with clinical and serological characteristics	Defines immune endotypes that predict prognosis and therapeutic responsiveness

Recognition of immune microenvironmental diversity has profound implications for the clinical management of MG. The differences in immune cell composition, cytokine profiles, complement activity, thymic pathology, and tissue-specific inflammation contribute to the substantial variability observed in MG severity and therapeutic response (15, 44). Patients with similar antibody titers may experience markedly different clinical outcomes because their underlying immune ecosystems differ in cellular organization and inflammatory activity (**Table 3**) (78–82). Advances in high-dimensional immunophenotyping, flow cytometry, mass cytometry, single-cell transcriptomics, proteomics, metabolomics, and spatial transcriptomics now provide unprecedented opportunities to characterize these immune landscapes at cellular resolution. Integration of these technologies with clinical phenotyping may enable identification of immune endotypes that more accurately predict prognosis and therapeutic responsiveness than conventional antibody classification alone (63, 83, 84). Thus, understanding the immune microenvironment shifts the conceptual framework of MG from a disorder defined solely by pathogenic antibodies to one governed by dynamic interactions among immune cells, inflammatory mediators, and tissue-specific niches (39). This systems-level perspective provides the foundation for precision medicine, where therapeutic selection is guided by an individual’s unique immunological landscape rather than by clinical phenotype alone.

**Table 3:** The limitation of the therapies targeting a single immune component in MG.

Therapeutic target	Primary mechanism	Major limitation	Potential combination/sequential strategy
Anti-CD20 B-cell depletion	Eliminates circulating CD20^+^ B cells and reduces antigen presentation	Does not eliminate long-lived plasma cells or existing autoantibodies	Sequential use with FcRn inhibitors or plasma cell-targeted therapies has been proposed based on complementary mechanisms, but clinical evidence in MG remains limited. Potential concerns include cumulative immunosuppression, increased infection risk, and hypogammaglobulinemia. Well-designed prospective studies are needed before routine implementation (85)
Complement inhibition	Prevents membrane attack complex formation and NMJ injury	Upstream autoantibody production persists	Combination with immunomodulatory therapies is biologically promising to control both downstream tissue injury and ongoing autoantibody production. However, evidence is largely derived from mechanistic rationale and clinical experience rather than controlled trials. Meningococcal vaccination and infection monitoring remain essential (81)
FcRn inhibition	Accelerates degradation of circulating IgG, including pathogenic autoantibodies	Does not affect autoreactive B cells, plasma cells, or thymic immune dysregulation	FcRn inhibitors may serve as rapid disease-control agents before or during maintenance immunotherapy, but optimal sequencing has not been established. Current evidence supports efficacy of FcRn blockade as monotherapy, while combination strategies require further evaluation for long-term safety and durability (86, 87)
Thymectomy	Removes a major source of autoreactive immune activation in AChR-positive MG	Peripheral immune memory and long-lived plasma cells may persist	Thymectomy is routinely performed with standard immunotherapy in appropriately selected patients, supported by randomized and long-term follow-up studies. This represents established multimodal management rather than an investigational combination strategy (88)
Conventional immunosuppression	Broad suppression of adaptive immune responses	Variable efficacy, delayed onset, and non-specific immunosuppression	Conventional immunosuppressants are frequently used alongside corticosteroids and increasingly with targeted biologics in refractory disease. Although this approach reflects contemporary clinical practice, comparative evidence defining optimal sequencing or combination regimens remains limited, and cumulative toxicity and infection risk should be carefully monitored (89)

## Heterogeneity in myasthenia gravis

Age at disease onset is a major determinant of MG heterogeneity and is closely associated with differences in immunopathology, clinical presentation, and therapeutic response. Traditionally, MG is classified into early-onset MG (EOMG) and late-onset MG (LOMG), which usually develops after the fifth decade of life. Early-onset MG predominantly affects women and is strongly associated with thymic follicular hyperplasia (72). The immune microenvironment in these patients is characterized by active germinal center formation, autoreactive B-cell proliferation, increased follicular helper T-cell activity, and robust autoantibody production. The thymectomy often provides significant long-term clinical benefit in carefully selected patients with EOMG (63). In contrast, late-onset MG occurs more frequently in men and develops in the context of immunosenescence, a process characterized by progressive deterioration of immune regulation with aging. Age-related reductions in naïve T-cell production, impaired Treg function, chronic low-grade inflammation, so-called inflammaging, and expansion of memory lymphocyte populations collectively alter immune homeostasis (90, 91). These changes may contribute to persistent autoimmunity while simultaneously increasing susceptibility to infections and adverse effects of immunosuppressive therapy. Clinically, patients with LOMG often exhibit greater disease severity, higher rates of generalized weakness, and increased comorbidity burden. Age-associated cardiovascular disease, diabetes mellitus, osteoporosis, and renal impairment complicate treatment selection and increase the risks associated with long-term corticosteroid therapy (92, 93). Therefore, chronological age should be considered alongside biological immune age when individualizing therapeutic strategies.

Sex differences are another important dimension of MG heterogeneity. Women are disproportionately affected during early adulthood, while the incidence in men increases substantially after the age of 50 years (94, 95). These differences reflect complex interactions among sex hormones, genetic factors, and immune regulation. Estrogen exerts pleiotropic effects on immune function by promoting B-cell survival, antibody production, and T-helper cell activation while influencing cytokine secretion. These immunostimulatory properties may partly explain the increased susceptibility of younger women to autoimmune diseases, such as MG (95). Conversely, testosterone generally suppresses inflammatory immune responses and promotes regulatory immune pathways, potentially providing partial protection against autoimmune activation in younger men (96, 97). Hormonal fluctuations also influence disease activity throughout life. Pregnancy induces profound immunological adaptations that may stabilize disease in some patients while increasing the risk of exacerbation during the postpartum period when immune homeostasis is rapidly restored. Menopause may similarly alter immune regulation through declining estrogen concentrations, although its effects on MG remain incompletely understood (98). Beyond hormonal influences, sex-specific genetic factors, including genes encoded on the X chromosome and epigenetic regulation of immune signaling pathways, likely contribute to differences in disease susceptibility and progression (96). Recognition of these biological differences may facilitate more individualized approaches to disease monitoring and therapeutic intervention.

## Geographic heterogeneity

Significant geographic and ethnic variation exists in the epidemiology and clinical characteristics of MG. Differences in disease prevalence, antibody distribution, thymic pathology, and clinical presentation have been reported across populations worldwide, suggesting that genetic background and environmental exposures jointly influence disease expression (99, 100). However, these observed differences should be interpreted cautiously, as they may also reflect variation in healthcare access, diagnostic criteria, disease awareness, referral patterns, and study methodology. Consequently, distinguishing true biological heterogeneity from methodological and healthcare-related confounders remains a major challenge in population-based MG research. East Asian populations demonstrate a relatively higher prevalence of ocular MG compared with Western populations, whereas early-onset disease appears particularly common in certain Asian countries (101, 102). Although these patterns may partly reflect population-specific genetic susceptibility, environmental factors, including infectious exposures, dietary habits, vitamin D status, and gut microbiome composition, have also been proposed to influence immune regulation and disease phenotype. In contrast, African and African-descendant populations have been reported to exhibit earlier disease onset, greater disease severity, and a higher frequency of treatment-refractory disease in some cohorts (103). These findings may be associated with differences in immunogenetic architecture, socioeconomic determinants of health, delays in diagnosis, and disparities in access to specialized neuromuscular care. Conversely, European populations show a higher proportion of thymoma-associated MG and late-onset disease, although substantial heterogeneity exists across countries and age groups, emphasizing that regional variation persists even within broadly defined ethnic populations (104).

Ethnic diversity also contributes to variation in immunogenetic susceptibility. Associations between MG and HLA alleles differ substantially among European, Asian, Middle Eastern, and African populations, reflecting population-specific patterns of linkage disequilibrium and evolutionary selection (105, 106). These variations affect antigen presentation, T-cell selection, and immune tolerance, thereby modifying disease susceptibility and potentially influencing therapeutic responsiveness. Despite these recognized differences, many pivotal clinical trials evaluating biological therapies have predominantly enrolled patients from Europe and North America. Therefore, evidence regarding treatment efficacy in underrepresented populations remains limited, highlighting an important gap in the development of globally applicable precision medicine strategies.

Population heterogeneity may also have important therapeutic implications. Nevertheless, robust comparative data across diverse populations remain limited. Most pivotal clinical trials evaluating biological therapies have predominantly enrolled patients from Europe and North America, resulting in the under-representation of many Asian, African, Latin American, and Middle Eastern populations. Consequently, the generalizability of efficacy, safety, and biomarker data to these populations remains uncertain. Expanding the geographic and ethnic diversity of future clinical trials, together with integrating genomic, environmental, immunological, and socioeconomic data, will be essential for identifying population-specific disease endotypes and advancing globally applicable precision medicine strategies for MG.

## Biomarkers and precision medicine in myasthenia gravis

Biomarkers remain the most widely used tools in the clinical evaluation of MG, extending beyond diagnosis to encompass disease susceptibility, prognosis, therapeutic selection, and treatment monitoring. Organizing biomarkers according to their clinical function provides a useful framework for understanding their current applications and future potential in precision medicine.

### Diagnostic biomarkers

Autoantibodies directed against AChR, MuSK, and LRP4 establish the diagnosis and provide important information regarding disease subtype and underlying immunopathology (107). AChR antibodies are detected in the majority of patients with generalized MG and are closely associated with complement-mediated destruction of the neuromuscular junction (48). In contrast, MuSK antibodies belong predominantly to the IgG4 subclass and disrupt neuromuscular signaling without significant complement activation. LRP4 antibodies define an additional subgroup with distinct clinical characteristics, although their pathogenic role remains less clearly understood (9).

### Susceptibility biomarkers

Genetic biomarkers primarily reflect disease susceptibility rather than disease activity. Multiple susceptibility loci associated with HLA genes and immune regulatory pathways contribute to MG risk and may partially explain differences in disease subtype and age at onset. Although susceptibility loci related to MG variants primarily indicate disease susceptibility rather than disease activity, integration of genomic information may eventually facilitate prediction of therapeutic responsiveness and long-term prognosis.

### Prognostic biomarkers

Despite their diagnostic importance, antibody titers correlate inconsistently with disease severity or treatment response. Some patients experience substantial clinical improvement despite persistently elevated antibody concentrations, whereas others develop severe weakness despite relatively modest titers (108). It indicates that circulating antibody levels alone are insufficient to characterize disease activity and should be interpreted alongside additional immunological biomarkers. Improvements in highly sensitive cell-based antibody detection methods continue to expand the spectrum of clinically relevant autoantibodies and may reduce the proportion of patients considered seronegative.

Transcriptomic analyses have revealed distinct gene expression profiles associated with different MG subtypes. Increased expression of interferon-responsive genes, inflammatory signaling pathways, and B-cell activation markers has been observed in patients with active disease (63, 109). Proteomics provides complementary information by quantifying circulating proteins involved in immune regulation, complement activation, extracellular matrix remodeling, and neuromuscular junction integrity (83). Similarly, metabolomic profiling has identified alterations in amino acid metabolism, lipid metabolism, oxidative stress pathways, and mitochondrial function that may reflect immune activation and tissue injury (110, 111).

### Predictive biomarkers

Predictive biomarkers are intended to identify patients most likely to respond to specific therapies. At present, MG subtype-specific autoantibodies provide the strongest clinically applicable predictive information because they reflect distinct pathogenic mechanisms that influence therapeutic responsiveness. For example, patients with AChR antibody-positive MG may derive greater benefit from complement inhibition, whereas MuSK antibody-positive disease exhibits distinct immunopathology characterized by IgG4-mediated dysfunction rather than complement activation. Beyond autoantibodies, transcriptomic profiling may eventually identify molecular signatures associated with responsiveness to targeted immunotherapies, although these findings require prospective validation before clinical implementation.

### Pharmacodynamic biomarkers

Pharmacodynamic biomarkers monitor biological responses to treatment and may facilitate assessment of therapeutic efficacy. Although reductions in circulating autoantibody levels are observed in some patients following immunotherapy, changes in antibody titers often correlate poorly with clinical improvement and therefore have limited value as standalone markers of treatment response. Consequently, there is growing interest in identifying dynamic biomarkers that better reflect immune modulation during therapy.

Single-cell RNA sequencing further enables characterization of individual immune cell populations, identifying transcriptionally distinct B-cell, T-cell, and myeloid-cell subsets that contribute to disease heterogeneity (15). The integration of spatial transcriptomics further extends these insights by preserving tissue architecture, thereby allowing characterization of cellular interactions within the thymus, lymphoid tissues, and neuromuscular junction. These technologies provide unprecedented opportunities to elucidate the spatial and functional organization of pathogenic immune responses.

## Challenges

Integration of these multi-omics datasets has the potential to generate comprehensive molecular signatures capable of distinguishing biologically distinct disease endotypes and predicting therapeutic responses with greater accuracy than individual biomarkers alone. Despite their considerable promise, several important challenges currently limit the clinical translation of these emerging technologies. Most single-cell and spatial omics studies in MG have been conducted in relatively small, highly selected patient cohorts because of the disease and the limited availability of high-quality thymic and neuromuscular tissue samples. In addition, technical variability arising from differences in tissue processing, sequencing platforms, library preparation protocols, computational pipelines, and batch effects can complicate cross-study comparisons and reduce reproducibility. Biological heterogeneity, including differences in disease subtype, disease duration, treatment exposure, age, sex, and comorbidities, further complicates interpretation of multi-omics datasets and may obscure disease-specific molecular signals.

Furthermore, translating complex multi-omics discoveries into clinically actionable biomarkers remains a substantial challenge. Candidate biomarkers identified through high-dimensional analyses often demonstrate limited reproducibility across independent cohorts and may not be readily applicable in routine clinical practice because of high costs, technical complexity, limited assay standardization, and the need for specialized bioinformatic expertise. Prospective longitudinal studies incorporating standardized sampling protocols, harmonized analytical pipelines, and external validation across geographically and ethnically diverse populations will therefore be essential to distinguish robust biological signals from technical artifacts. Ultimately, successful implementation of multi-omics in precision medicine will depend on technological advances as well as on the development of clinically accessible, reproducible, and cost-effective biomarkers that can guide patient stratification and therapeutic decision-making.

## Conclusion

A central premise of this review is that the immune microenvironment represents a critical biological interface through which genetic susceptibility, antibody subtype, age, sex, ethnicity, thymic pathology, and environmental exposures converge to generate the remarkable clinical and immunological heterogeneity observed in MG. Unlike previous reviews that have primarily focused on individual immunological pathways or emerging therapeutic agents, we integrate advances in immune microenvironment biology with population heterogeneity and multi-omics technologies to provide a unified framework for understanding disease endotypes and their implications for precision therapeutics. By critically evaluating how immune-cell interactions, tissue-specific immune niches, and host-specific factors collectively influence disease manifestations and therapeutic responses, this review highlights why patients with similar serological profiles frequently exhibit divergent clinical trajectories and variable responses to targeted therapies.

This integrated perspective underscores that MG should be viewed as a spectrum of biologically distinct immune endotypes rather than a single antibody-mediated disease. We propose that future precision medicine strategies should incorporate both molecular and population-level heterogeneity to improve patient stratification, optimize therapeutic selection, and facilitate the development of individualized treatment algorithms. Ultimately, bridging mechanistic immunology with clinically validated biomarkers and inclusive, globally representative translational research will be essential for transforming advances in immune microenvironment research into durable, personalized therapies for patients with MG.
